# Good Performance of the Chinese Version of Mini Social Cognition and Emotional Assessment in the Early Diagnosis of Behavioral Variant Frontotemporal Dementia

**DOI:** 10.3389/fneur.2022.827945

**Published:** 2022-02-17

**Authors:** Fen Wang, Aihong Zhou, Cuibai Wei, Xiumei Zuo, Xiaowei Ma, Lina Zhao, Hongmei Jin, Yan Li, Dongmei Guo, Jianping Jia

**Affiliations:** ^1^Department of Neurology, Innovation Center for Neurological Disorders, Xuanwu Hospital, Capital Medical University, Beijing, China; ^2^Department of Neurology, The First Hospital of Hebei Medical University, Shijiazhuang, China

**Keywords:** mini-SEA, social cognition, behavioral variant frontotemporal dementia, Alzheimer's disease, early diagnosis

## Abstract

Social cognition impairment has been recognized as an early and characteristic change in behavioral variant frontotemporal dementia (bvFTD). The Mini Social Cognition and Emotional Assessment (mini-SEA) is a clinical tool to rapidly evaluate social cognition. In this study, we explored the diagnostic value of social cognition by assessing the Chinese version of the mini-SEA and other standard neuropsychological tests in 22 patients with mild bvFTD, 26 patients with mild Alzheimer's disease (AD), including mild cognitive impairment (MCI) and mild dementia, and 30 control subjects. The discriminatory powers of these tests were evaluated and compared using the receiver operating characteristic curve (ROC). The mini-SEA scores of the bvFTD patients were significantly lower than those of the controls (*Z* = –6.850, adjusted *P* < 0.001) and AD patients (*Z* = –3.737, adjusted *P* = 0.001). ROC analysis showed that the mini-SEA had a high discriminatory power for differentiating bvFTD from the controls, with an area under the curve (AUC) value of 0.989 (95% *CI* = 0.905-1.000, *P* < 0.001). The AUC value of the mini-SEA for differentiating bvFTD from AD was 0.899 (95% *CI* = 0.777-0.967, *P* < 0.001), higher than that of the Auditory Verbal Learning Test Delayed Recall (AUC = 0.793), Boston Naming Test (AUC = 0.685) or Frontal Assessment Battery (AUC = 0.691). The Chinese version of mini-SEA is a good clinical tool for the early diagnosis of bvFTD, and has a high sensitivity and specificity to discriminate bvFTD from AD.

## Introduction

Behavioral variant frontotemporal dementia (bvFTD) is a pathologically and genetically heterogeneous neurodegenerative disorder characterized by progressive behavioral abnormalities, personality changes and impaired social interaction (1). It is a leading cause of early-onset neurodegenerative dementia along with Alzheimer's disease (AD) (2). Due to the absence of definitive biomarkers, bvFTD is difficult to identify accurately in the early stages and may be underdiagnosed or misdiagnosed as AD, depression, or other psychiatric disorders (3). In 2011, the International Behavioral Variant Frontotemporal Dementia Criteria Consortium (FTDC) revised the diagnostic criteria of bvFTD, and classified the diagnosis into three levels: possible, probable and definite bvFTD (4). The diagnostic criteria are sensitive and practical, but accurate diagnosis of bvFTD remains to be improved (5). Many research showed that the criteria on the neuropsychological profile (F: executive deficits with relative sparing of memory) might not be optimal. The application of traditional executive function tests in the early and differential diagnosis of bvFTD has yielded inconsistent results (6, 7). Recent studies also reported that large impairment in memory also occurred in patients with bvFTD and should not be regarded as an exclusion criterion (8). More and more researchers are turning their attention to social cognition testing in order to improve the criteria of bvFTD.

Social cognition is the ability to perceive and interpret information about other people and social situations, and plays a key role in social behaviors and interpersonal communication. It is usually thought to include theory of mind (ToM), emotion recognition, empathy, social knowledge and insight (9). The neural substrate for social cognition is medial prefrontal cortex (mPFC), orbitofrontal cortex, anterior cingulate cortex, insula and amygdala, etc. (10), which are obviously affected early in bvFTD (11). Therefore, social cognition impairment has been thought to be an early and characteristic change in bvFTD, and many cognitive tests for social cognition are being actively developed and applied in the western population (9). Social cognition is greatly influenced by culture (12). Anthropologist and psychologists have long known that individuals raised in different cultures, East and West, have different understandings of themselves and their relationships with others, referred as the “collectivism” and “individualism” (13). Both emotion recognition and expression are related to culture, and facial expressions are easier to recognize by members of the same ethnic or national group (14). Moreover, neural correlates of ToM may also differ across cultures (15), and medial prefrontal cortex, the key structure of the social neural network, is suggested to be employed in a culture-dependent manner (13). Therefore, it is critical to develop culturally appropriate clinical tools to evaluate social cognition.

The Mini Social Cognition and Emotional Assessment (mini-SEA) is a tool to rapidly evaluate ToM and emotion recognition clinically (16). It has been considered as a promising diagnosis tool for bvFTD in the early stages and reported to have a good sensitivity and specificity to distinguish bvFTD from AD (17). However, the mini-SEA, developed in the western cultural context, may not suitable for Chinese bvFTD patients, given the striking cultural differences in social cognition. The adaptation of the mini-SEA will help us to investigate social cognition in Chinese population, and verify whether clinical features of bvFTD are comparable across different ethnicities. In this study, we used the Chinese version of mini-SEA and other standard neuropsychological tests in patients with mild bvFTD and AD, and aim to (1) assess the social cognition profile and other cognition domains (executive function, episodic memory, language, etc.); (2) explore the value of the Chinese version of mini-SEA for the early diagnosis of bvFTD and its ability to discriminate bvFTD from AD.

## Materials and Methods

### Participants

Seventy-eight participants including 22 patients with mild bvFTD, 26 patients with mild AD and 30 control subjects were recruited from January 2018 to December 2019. All of the patients (bvFTD and AD) were enrolled from the memory clinic of Xuanwu Hospital and each patient and his/her guardian received a semi-structured interview collecting detailed demographic data and medical history. All patients underwent a standard physical and neurological examination, a neuropsychological test battery, blood tests (blood routine, liver and kidney functions, serum levels of electrolyte, ammonia, homocysteine, folic acid, vitamin B12, and thyroid hormone, syphilis and AIDS antibody) and brain conventional magnetic resonance imaging (MRI) scan. 18F-Fluorodeoxyglucose positron emission tomography (18F-FDG PET) scans were performed for some bvFTD and AD patients to reveal the frontotemporal or temporoparietal hypoperfusion. To further clarify the diagnosis, some patients also underwent cerebrospinal fluid (CSF) examination to test p-Tau and Aβ-42 level, and/or Amyloid-PET, and/or related genetic testing. The final diagnosis of each patient was approved by two clinical experts on dementia. In order to improve the diagnostic accuracy, all patients were followed up for 6–12 months.

The patients with bvFTD met the revised diagnostic criteria for the probable or definite bvFTD proposed by FTDC in 2011 (4). All the patients had progressive deterioration of behavioral and/or cognition with the Mini-Mental State Examination (MMSE) scored >20/30, Clinical Dementia Rating (CDR) scored 0.5 or 1, meeting the requirements for the mild stage of bvFTD established in this study. Brain MRI and/or FDG-PET showed frontotemporal atrophy and/or hypoperfusion. Some patients underwent the lumbar puncture (*n* = 12) and amyloid-PET (*n* = 3) to excluded AD and other types of dementia. Genetic testing was performed in 11 patients with bvFTD. Two of them carried *MAPT* mutation (P513A and P301L) and one carried *GRN* mutation (S106R). Fourteen patients with bvFTD did not take any drugs before enrollment. Five patients have treated with serotonin reuptake inhibitors, two patients with Donepezil, and one patient has taken olanzapine.

The patients with AD met the revised diagnostic criteria for mild cognitive impairment (MCI) due to AD (*n* = 15) or probable AD dementia (*n* = 11) proposed by National Institute on Aging and Alzheimer's association (NIA-AA) in 2011 (18, 19). All the AD patients, including MCI or mild dementia (MD), had progressive episodic memory loss with MMSE scored >20/30 and CDR scored 0.5 or 1. Brain MRI and/or FDG-PET showed hippocampal atrophy and/or medial temporal hypoperfusion. All the patients with MCI due to AD had the evidence of amyloid deposition (elevated CSF p-Tau/Aβ-42 and/or positive amyloid-PET). None of the AD patients received any antidepressant, antipsychotic, or antidementia drugs before enrollment.

The patients were excluded if they met one of the following items: (1) vascular cognitive impairment supported by medical history and/or MRI findings; (2) clinically predominant aphasia; (2) motor neuron disease; (3) inflammatory, metabolic and other related disorders that cause cognitive impairment; (4) severe visual and auditory impairment interfering the cognitive assessment.

The gender- and education-matched healthy control subjects were recruited from the patients' spouse or healthy elderly at physical examination center of Xuanwu Hospital. The controls were included in the study according to the following criteria: (1) MMSE scored ≥ 27/30, CDR = 0 and Frontal Assessment Battery (FAB) ≥ 16/18; (2) No memory complaints or behavior problems or cognitive impairment; (3) No history of neurological or psychiatric illness; (4) No severe visual and auditory impairment interfering the cognitive assessment.

This study was approved by the Institutional Ethical Committee of Xuanwu Hospital. Informed consent was obtained from each subject either directly or indirectly from his or her guardian.

### Neuropsychological Background Tests

Both case and control subjects completed the Chinese version of MMSE (20), CDR (21), and FAB (22) to assess the global cognition and frontal lobe function. The bvFTD and AD patients also received a battery of neuropsychological tests to assess the: (1) Executive function: the Trail Making Test A (TMT-A) and B (TMT-B) (23), Digit Span Test forward (DST-F) and backward (DST-B) (24); (2) Episodic memory: modified World Health Organization-University of California Los Angeles Auditory Verbal Learning Test Immediate Recall (AVLT-I) and Delayed Recall (AVLT-D) (25); (3) Language: Boston Naming Test (BNT) (26), and Animal Fluency Test (AFT) (27); (4) Neuropsychiatric status: Frontal Behavioral Inventory (FBI) (28) and Geriatric Depression Scale (GDS) (29).

### Mini Social Cognition and Emotional Assessment (Mini-SEA)

All participants undertook the Chinese version of the mini-SEA to evaluate the social cognition and emotion performance. The English version of the mini-SEA (16), provided and authorized by professor Bertoux., was translated and adapted into Chinese (mandarin) by two neurologists (W.F., Z.A.) and one psychologist (Z.L.). Then, the Chinese draft was translated back into English by another independent psychologist working in an English-speaking country, and the back translation version was sent to Professor Bertoux. After full communication with Professor Bertoux, the Chinese version of the mini-SEA was established.

The mini-SEA is composed of two parts, a shortened version of the Faux-Pas test (FPT) and a reduced facial emotion recognition test (FERT). In the FPT, 10 social scenes (+1 example scene) are presented, including 5 faux-pas stories (scored from 0 to 30) and 5 control stories without a faux pas (scored from 0 to 10). Patients were required to detect and explain the social inconveniences in the stories. Some adaptations were required according the Chinese cultural: (1) The English name in all the stories was changed to Chinese name; (2) The “blonde hair” in story 4 was changed to “black hair”; (3) The dialogue at the end of story 5 was modified to the following sentence: Her boyfriend said “Never mind. Don't give up. You can get an opportunity next time.” She said: “Alright, I will keep studying hard.”; (4) “The book he wanted about hiking in the Grand Canyon” in story 6 was changed to “a travel book about Beijing”; (5) “apple pie” in story 7 was changed to “apple cake”. In the FERT, 35 faces with 7 different facial emotions (happiness, surprise, neutral, sadness, fear, disgust, and anger) are presented (scored from 0 to 35). Patients were required to recognize emotions of faces. The 35 face stimuli in the Chinese version of mini-SEA have been selected from the Chinese Facial Affective Picture System (CFAPS) to match the 35 white faces from the original FERT in terms of intensity, age, emotions and gender (30). The total FPT and FERT scores then converted to the composite subscores (scored from 0 to 15 respectively). The overall mini-SEA composite score was obtained by adding the two composite subscores (scored from 0 to 30).

The Chinese version of mini-SEA showed good validity and reliability. Validity was assessed by content validity index (CVI). Three experts were invited to evaluate each story and each picture. The S-CVI/UA (scale-level CVI based on the universal agreement method) were 0.83, and the S-CVI/Ave (scale-level CVI based on the average method) was 0.94. Reliability was assessed by intraclass correlation coefficient (ICC). Inter-rater reliability was performed on 30 subjects, including 10 bvFTD patients, 10 AD patients, and 10 controls. All subjects assessed by two raters simultaneously. For half of them, rater 1 asked questions, while rater 2 looked on and scored their performance. For the other half, rater 1 and rater 2 switched their roles. All subjects were re-evaluated by the rater who asked questions in the first rating after 4 weeks. The test-retest reliability was ICC 0.85 for the mini-SEA, and the inter-rater reliability was 0.92. The Cronbach's alpha coefficient was 0.71 for the FPT, 0.87 for the FERT, and 0.74 for the mini-SEA. The Chinese version of mini-SEA in this study were completed by two experienced well-trained raters, who received a standardized training. Mini-SEA was scored blind to the other instruments.

### Statistical Analysis

The normality of the demographic and neuropsychological data for the three groups was tested using Shapiro-Wilk method. To facilitate comparison with previous research results, all data were expressed as mean ± standard deviation (SD). Parametric data (age at visit and education) were analyzed across three groups by analysis of variance (ANOVA), followed by *post-hoc* tests for pairwise comparison (Scheffe method). The student's *t*-test was used to compare means of age at onset and duration between bvFTD and AD group. If the normal distribution is not satisfied (neuropsychological data), Kruskal-Wallis test was used to compare the three groups, followed by Mann-Whitney test for two-by-two comparisons. Bonferroni correction was used for multiple comparisons. The adjusted *P*-value was obtained by multiplying the original *P*-value by the number of comparisons (*N* = 3). Differences in gender among three groups were assessed by the Pearson Chi-square test. These statistical analyses were performed using SPSS 20.0 software.

Receiver operating characteristics (ROC) curves were used to evaluate the discriminatory power of each test by calculating the area under the curve (AUC). Cut-off points were determined using Youden's index to select the point giving best sensitivity and specificity. The difference between two ROC curves were calculated by a nonparametric method (Delong's method). The ROC analyses were performed by MedCalc Statistical Software 18.2.1. All statistical tests were two sided, and *P* < 0.05 was considered statistically significant.

## Results

### Demographic Data and General Neuropsychological Tests

The demographic data of bvFTD, AD (MCI+MD) and control groups are shown in Table 1. There were no statistically significant differences in gender and education level among the three groups (all *P* > 0.05). The mean age of the bvFTD patients was not statistically different from that of the controls (*P* > 0.05), but the AD patients was significantly older than the bvFTD patients (69.15 *vs*. 62.95, *P* = 0.027) or controls (69.15 *vs*. 62.93, *P* = 0.015). The mean age of onset of the bvFTD group was significantly earlier than that of the AD (MCI+MD) group (59.77 *vs*. 66.92, *P* = 0.007).

**Table 1:** Demographic characteristics and general neuropsychological test data for the bvFTD, AD and control groups.

**Characteristics**	**bvFTD *N =* 22**	**AD (MCI+MD) *N =* 26**	**Controls *N =* 30**	**bvFTD vs. AD**	**bvFTD vs. Controls**	**AD vs. Controls**
Gender (F/M)	11/11	14/12	17/13	NS	NS	NS
Age at visit, years	62.95 ± 8.59	69.15 ± 8.50	62.93 ± 6.36	*P* = 0.027	NS	*P* = 0.015
Age at onset, years	59.77 ± 9.00	66.92 ± 8.65	−	*P* = 0.007	–	–
Duration, months	29.05 ± 17.91	26.85 ± 16.08	−	NS	–	–
Education, years	12.18 ± 3.29	10.92 ± 3.16	11.80 ± 3.04	NS	NS	NS
MMSE	24.23 ± 3.25	23.54 ± 2.01	29.07 ± 1.08	NS	*P* < 0.001	*P* < 0.001
FAB	14.14 ± 3.09	16.08 ± 1.41	17.50 ± 0.63	NS	*P* < 0.001	*P* = 0.002
CDR-SOB	4.00 ± 1.26	3.00 ± 1.20	0	NS	*P* < 0.001	*P* < 0.001
FBI	26.95 ± 8.87	7.23 ± 4.11	−	*P* < 0.001	–	–
GDS	6.18 ± 6.29	6.04 ± 4.45	−	NS	–	–

*Data expressed as number of subjects or mean ± SD*.

*MMSE, mini-mental state examination; FAB, frontal assessment battery; CDR, clinical dementia rating; SOB, sum of boxes; FBI, frontal behavioral inventory; GDS, geriatric depression scale; NS, not significant*.

Compared with the control group, MMSE and FAB scores were significantly worse in the bvFTD (*Z* = −5.208, adjusted *P* < 0.001 and *Z* = –4.919, adjusted *P* < 0.001, respectively) and AD patients (*Z* = –6.132, adjusted *P* < 0.001 and *Z* = –3.426, adjusted *P* = 0.002, respectively). However, MMSE scores did not significantly differ between the bvFTD and AD (MCI+MD) group (*P* > 0.05). The bvFTD patients had lower FAB scores than AD, suggesting more frontal dysfunction in bvFTD, though the differences did not reach statistical significance (*Z* = –1.597, adjusted *P* = 0.331). Not surprisingly, worse performance of the FBI tests was seen in bvFTD than AD (*Z* = 5.813, *P* < 0.001). No significant difference was observed in GDS scores between the bvFTD and AD (MCI+MD) groups (*P* > 0.05).

### Performance of the Mini-SEA and Other Cognitive Tests

The mini-SEA scores were significantly different among bvFTD, AD (MCI+MD) and control groups (*H* = 46.940, *P* < 0.001), as well as scores of FTP (*H* = 37.749, *P* < 0.001) and FERT (*H* = 36.406, *P* < 0.001) (Table 2). Pairwise comparison showed that the mini-SEA scores of the bvFTD group were significantly worse than those of the control group (*Z* = –6.850, adjusted *P* < 0.001) and AD (MCI+MD) group (*Z* = –3.737, adjusted *P* = 0.001). The bvFTD patients also showed significantly more damage on both the FPT and FERT than controls (*Z* = –6.092, adjusted *P* < 0.001 and *Z* = –6.030, adjusted *P* < 0.001, respectively) and AD patients (*Z* = –4.070, adjusted *P* < 0.001 and *Z* = –3.185, adjusted *P* = 0.004, respectively). The FERT and mini-SEA tests was slightly impaired in the AD (MCI+MD) group compared with the control group (*Z* =-2.873, adjusted *P* = 0.012 and *Z* = –3.136, adjusted *P* = 0.005, respectively) and there was no difference on the FPT scores between the two groups (*P* > 0.05). After controlling the age, the statistical results on the mini-SEA were not affected.

**Table 2:** Performance of Mini-SEA and cognitive tests on executive function, episodic memory and language for the bvFTD, AD and control groups.

**Tests**	**bvFTD *N =* 22**	**AD (MCI+MD) *N =* 26**	**Controls *N =* 30**	**bvFTD vs. AD**	**bvFTD vs. Controls**	**AD vs. Controls**
mini-SEA	15.79 ± 3.77	21.86 ± 2.14	24.27 ± 1.90	*P* = 0.001	*P* < 0.001	*P* = 0.005
FPT	7.56 ± 2.45	11.34 ± 1.64	12.40 ±1.48	*P* < 0.001	*P* < 0.001	NS
FERT	8.24 ± 2.02	10.52 ± 1.56	11.87 ± 1.04	*P* = 0.004	*P* < 0.001	*P* = 0.012
**Executive function**						
TMT-A	77.88 ± 31.22	68.15 ± 28.50	–	NS	–	–
TMT-B	190.00 ± 84.36	168.65 ± 86.31	–	NS	–	–
DST-F	7.41 ± 1.33	7.58 ± 1.10	–	NS	–	–
DST-B	4.24 ± 1.48	3.96 ± 0.96	–	NS	–	–
**Episodic memory**						
AVLT-I	18.10 ± 6.20	16.42 ± 4.49	–	NS	–	–
AVLT-D	4.43 ± 3.23	1.19 ± 2.14	–	*P* < 0.001	–	–
Language						
BNT	18.63 ± 5.20	21.62 ± 4.16	–	*P* = 0.046	–	–
AFT	11.20 ± 4.23	12.19 ± 3.52	–	NS	–	–

*Data expressed as mean ± SD*.

*mini-SEA, the abbreviated version of the social cognition and emotional assessment; FPT, faux-pas test; FERT, facial emotion recognition test; TMT, trail making test; DST, digit span test; AVLT, auditory verbal learning test; BNT, boston naming test; AFT, animal fluency test; NS, not significant*.

There were no statistically significant differences in TMT-A, TMT-B, DST-F, DST-B, AVLT-I, and AFT scores between the bvFTD and AD (MCI+MD) groups (all *P* > 0.05). However, bvFTD patients significantly performed better than AD patients on the AVLT-D test (*Z* = 3.597, *P* < 0.001), and worse on the BNT test (*Z* = –2.000, *P* = 0.046). After controlling the age, the statistical results on the neuropsychological tests were not affected.

### ROC Analysis for the Mini-SEA, FPT and FERT

ROC analysis (Table 3) showed that the mini-SEA had a high discriminatory power for differentiating the bvFTD patients from the controls, with an AUC value of 0.989 (95% *CI* = 0.905–1.000, *P* < 0.001), as well as FPT (AUC = 0.954, 95% *CI* = 0.857–0.993, *P* < 0.001) and FERT (AUC = 0.953, 95% *CI* = 0.856–0.992, *P* < 0.001). The sensitivity and specificity were 95.5% and 93.3% respectively with a cut-off value at 21.4 for the mini-SEA.

**Table 3:** ROC analysis for Mini-SEA, FPT and FERT to discriminate the bvFTD group from the controls or AD group.

**Tests**	**Groups**	**AUC**	**95% *CI***	**SE**	***P* value**	**Cutoff**	**Sensitivity**	**Specificity**
**mini-SEA**	bvFTD *vs*. Controls	0.986	0.905–1.000	0.011	<0.001	≤ 21.4	0.955	0.933
	bvFTD *vs*. AD	0.899	0.777–0.967	0.049	<0.001	≤ 18.9	0.818	0.962
**FPT**	bvFTD *vs*. Controls	0.954	0.857–0.993	0.028	<0.001	≤ 9.8	0.864	0.900
	bvFTD *vs*. AD	0.891	0.767–0.962	0.052	<0.001	≤ 9.8	0.864	0.846
**FERT**	bvFTD *vs*. Controls	0.953	0.856–0.992	0.028	<0.001	≤ 10.3	0.909	0.900
	bvFTD *vs*. AD	0.809	0.670–0.908	0.062	<0.001	≤ 9.4	0.773	0.692

*ROC, receiver operating characteristics; mini-SEA, the abbreviated version of the social cognition and emotional assessment; FPT, faux-pas test; FERT, facial emotion recognition test; AUC, area under the curve; CI, confidence interval; SE, standard error*.

The AUC value of the mini-SEA for differentiating bvFTD from AD was 0.899 (95% *CI* = 0.777–0.967, *P* < 0.001), with 81.8% sensitivity and 96.2% specificity at a cut-off value of 18.9. However, the sensitivity and specificity of FERT (cutoff = 9.4) were <80%, obviously lower than that of FPT and mini-SEA, though the difference in AUC values was not statistically significant (*P* > 0.05).

### Comparisons of ROC Curves for the Mini-SEA, AVLT-D, BNT and FAB

**Figure 1:**
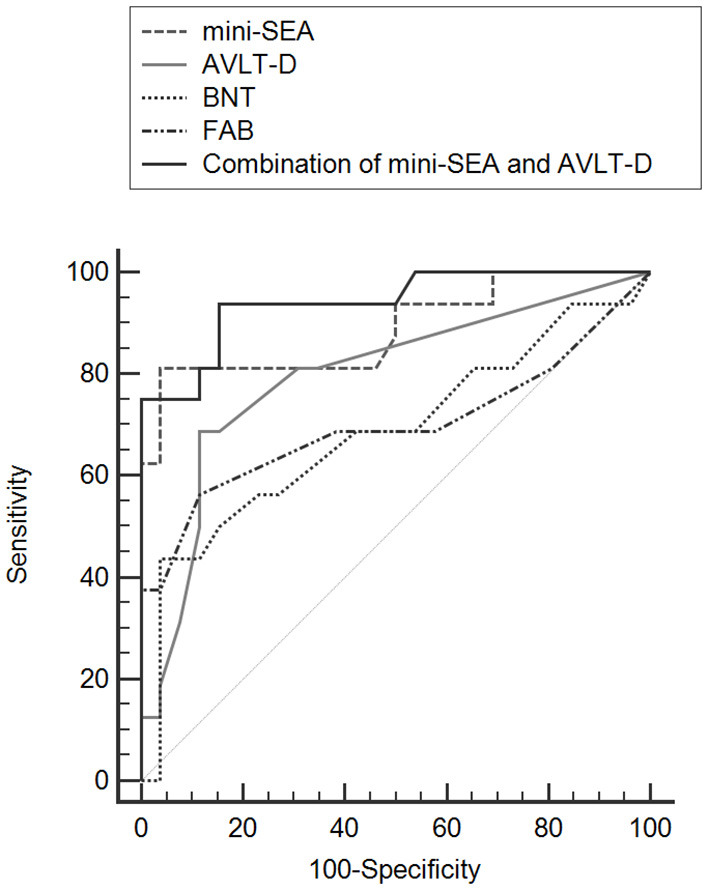
Comparisons of ROC curves for mini-SEA, AVLT-D, BNT, and FAB for discriminating bvFTD from AD.

The AUC values of the AVLT-D, BNT and FAB for differentiating bvFTD from AD was 0.793 (95% *CI* = 0.650–0.897, *P* < 0.001), 0.685 (95% *CI* = 0.524–0.819, *P* = 0.046) and 0.691 (95% *CI* = 0.540–0.818, *P* = 0.026) (Figure 1). The AUC value of the mini-SEA was higher than that of the AVLT-D, BNT and FAB, though the differences were statistically significant only between the mini-SEA and BNT (0.899 *vs*. 0.685, *Z* = 2.249, *P* = 0.025). Then, we combined the mini-SEA and AVLT-D, two tests with AUC values higher than 0.7. The discriminatory power of the new indicator was significantly better than that of AVLT-D (0.946 *vs*. 0.793, *Z* = 2.333, *P* = 0.020), BNT (0.946 *vs*. 0.685, *Z* = 2.690, *P* = 0.007) and FAB (0.946 *vs*. 0.691, *Z* = 2.283, *P* = 0.022).

## Discussion

This study established strict inclusion and exclusion criteria in accordance with internationally recognized diagnostic criteria (4), and all the enrolled patients were in the mild stage of the disease. The bvFTD group was mostly based on the clinical diagnosis, and only 3 patients diagnosed with evidence of gene mutations. The AD group included MCI individuals with evidence of amyloid deposition and mild dementia patients. We modified the English version of the mini-SEA by adopting Chinese facial affective pictures in FERT and making some adaptations to the stories of FPT according the characteristics of Chinese cultural. The results showed that both bvFTD and AD had social cognitive impairment, involving ToM and emotional recognition, in the early stages. However, the damages in bvFTD were more prominent and extensive than those in AD. Consistent with Bertoux's results (17), the Chinese version of mini-SEA had good sensitivity and specificity for differentiating mild bvFTD from controls or mild AD. To our knowledge, this is the first study to evaluate the social cognitive impairment of bvFTD in Chinese population. Our results suggests that clinical features of bvFTD are comparable across different ethnicities, and the Chinese version of mini-SEA can be used as an effective tool for early diagnosis of bvFTD in Chinese population. It is worth mentioning that the average scores of the Chinese version of mini-SEA in the control group were slightly lower than those of Bertoux's studies (24.3 *vs*. 25.8), and this trend was also seen in the both bvFTD and AD group. The discrepancy may come from cultural differences among ethnic groups. Cross-cultural research is necessary to understand social cognitive in health and neurodegenerative cognitive disorders.

The mini-SEA is composed of FPT and FERT, two tests on ToM and emotion recognition respectively. Then, the two subscores of mini-SEA were further analyzed in this study. The patients with bvFTD showed more significant impairment on the FPT than AD patients and controls, while no difference was seen between AD patients and controls. This was similar to what previous studies had reported. In 2002, Gregory et al. (31) first assessed ToM performance in patients with bvFTD and AD. They found that bvFTD patients had significant deficits on the FPT and other ToM tests, which closely correlated to the ventromedial frontal atrophy, while AD patients only showed deficits on memory-based questions. Subsequent research confirmed this idea (32, 33) and showed the FPT was not only a sensitive diagnostic indicator in early bvFTD than other cognitive measures (34, 35), but also a specific method that distinguished bvFTD from AD (36, 37). However, a recent longitudinal multicenter study did not find the difference on the baseline FPT scores between bvFTD and other neurodegenerative diseases (38). More longitudinal studies with larger samples are needed to confirm the diagnostic specificity of the FPT. Another important aspect of social cognition is emotion recognition, and FERT is one of the most commonly used tasks to assess it. Previous studies have shown that FERT scores in bvFTD patients were significantly lower than those in both control subjects (39–43) and AD patients (44–46). Meanwhile, facial emotion recognition was slightly impaired in early AD compare with controls, and progressively declined over the course of the disease (45). Meta-analysis showed that facial emotion recognition was significantly impaired in bvFTD, especially in negative emotions including anger, disgust, fear and sadness (47). The results of our study were consistent with the findings of previous studies, supporting the idea that bvFTD affected the facial emotion recognition in the early stage, and more profound impairment in emotion recognition were presented in bvFTD than that in AD patients.

In this study, we also assessed executive function, episodic memory and language in patients with bvFTD and AD. It has been proposed in the current diagnostic criteria that executive dysfunction was the core cognitive deficit in bvFTD. However, it is sometimes difficult to accurately distinguish AD from bvFTD using traditional executive function tests even in the early stages of the disease (6, 7). Consistent with previous studies, our results showed that there were no statistically significant differences in the TMT, DST and FAB tests between the two diseases. We found that the AVLT-D score in bvFTD was significantly higher than that in AD. It is generally accepted that episodic memory is relatively preserved in the early stages of bvFTD, though recent studies have shown that memory impairment also occur in the early stage of bvFTD (8, 48). Longitudinal results showed that measures on executive function and memory strongly overlapped for bvFTD and AD, thus did not accurately discriminate the two diseases (49). Previous studies showed that scores of the mini-SEA was correlated with gray matter volume within the medial prefrontal cortex (mPFC) (50, 51). Meanwhile, the atrophy in the mPFC is the most characteristic neuroanatomical changes in the early stage of bvFTD, and helps distinguish bvFTD from other types of neurodegenerative dementia. Therefore, social cognitive impairment is considered as crucial cognitive signatures of bvFTD, just as episodic memory deficits are the core symptom of AD. Supporting this view, the mini-SEA showed a better discriminatory power comparing with AVLT-D, BNT or FAB in this study, and a combination of tests for mini-SEA and AVLT-D might have the greatest ability to discriminate bvFTD from AD.

There are several limitations in this study. First, the sample size was small, which would influence the research outcomes. However, our samples can provide a power of 90% to detect the difference in the mini-SEA scores between bvFTD and AD, and a power of 99% to detect the diagnostic values of ROC curves for the mini-SEA at a significance level of 0.05. Second, AD patients were not matched on age with the bvFTD patients and controls. Since the age at onset of bvFTD is earlier than that of AD, this problem is likely to occur if patients are continuously collected over a relatively short period of time. Fortunately, the statistical results on neuropsychological data were not affected after controlling the age. Previous studies have shown that ToM and facial emotion recognition got worse with age (52, 53). Therefore, the older age in the AD patients might decreased the mini-SEA scores, but did not substantially affect the conclusion of our study. Third, not all patient's diagnoses were supported by evidence of CSF/PET biomarkers. Only the patients diagnosed as MCI due to AD or bvFTD with memory loss underwent the lumber puncture for AD biomarker measures or amyloid-PET to confirm or exclude AD. Last, social cognitive is impaired in many other diseases, such as schizophrenia, depression, Parkinson's disease, Huntington's disease, etc. Therefore, future studies with larger sample size, more types of disease and multiple biomarkers are needed to confirm the diagnostic value of the Chinese version of mini-SEA in bvFTD.

## Conclusion

This study revealed that compared with AD, bvFTD had more significant social cognitive impairment and relatively retained memory in the early stage of the disease. The Chinese version of mini-SEA is a good clinical tool for the early diagnosis of bvFTD, and has a high sensitivity and specificity to discriminate bvFTD from AD.

## Data Availability Statement

The original contributions presented in the study are included in the article/supplementary material, further inquiries can be directed to the corresponding author/s.

## Ethics Statement

The studies involving human participants were reviewed and approved by the Institutional Ethical Committee of Xuanwu Hospital. The patients/participants provided their written informed consent to participate in this study.

## Author Contributions

FW and JJ: study concept and design. FW, AZ, CW, XZ, and XM: acquisition of clinical data. LZ and HJ: acquisition of neuropsychological data. FW and YL: analysis of data and statistical analysis. FW, AZ, CW, XZ, DG, and JJ: drafted or revised the manuscript. FW: acquired financial support. All authors contributed to the article and approved the submitted version.

## Funding

This work was supported by the grants from the National Natural Science Foundation of China (No. 81100797) and Beijing high-level health talents training project (No. 2015-3-068).

## Conflict of Interest

The authors declare that the research was conducted in the absence of any commercial or financial relationships that could be construed as a potential conflict of interest.

## Publisher's Note

All claims expressed in this article are solely those of the authors and do not necessarily represent those of their affiliated organizations, or those of the publisher, the editors and the reviewers. Any product that may be evaluated in this article, or claim that may be made by its manufacturer, is not guaranteed or endorsed by the publisher.
